# ID2 attenuates post-MI ventricular arrhythmias by targeting GATA4 suppression to preserve mitochondrial function and Na^+^/K^+^-ATPase activity

**DOI:** 10.1515/med-2026-1518

**Published:** 2026-09-22

**Authors:** Gang Pan, Shihao Huang, Xuewen Wang, Mingxin Liu, Yuanhua Xu

**Affiliations:** Department of Cardiology, Yueyang Central Hospital, Yueyang, Hunan Province, China; Department of Cardiology, Yueyang Central Hospital, Teaching Hospital of Jishou University, Yueyang, Hunan Province, China

**Keywords:** myocardial infarction, ventricular arrhythmia, ID2, GATA4, mitochondrial dysfunction

## Abstract

**Objectives:**

Post-myocardial infarction (MI) ventricular arrhythmia (VA) remains a leading cause of sudden cardiac death. Current antiarrhythmic drugs often fail to target the core metabolic-structural remodeling underlying arrhythmias. The transcriptional repressor ID2, which has been implicated in cardiovascular development, remains poorly characterized regarding its role and contribution to VA pathogenesis. This study investigated the role of the transcriptional repressor ID2 in post-myocardial infarction ventricular arrhythmia.

**Methods:**

An MI rat model was established via left anterior descending (LAD) coronary artery ligation. ID2 expression in myocardial tissues was quantified by immunofluorescence and western blot. Flow cytometry and biochemical assays were employed to evaluate the impact of ID2 modulation on arrhythmogenesis. Hypoxia-challenged cardiomyocytes treated with rotenone (Complex I inhibitor) and digoxin (Na^+^/K^+^-ATPase inhibitor) were used to investigate ID2’s effects on mitochondrial dysfunction and Na^+^/K^+^-ATPase activity. Mechanistic studies of ID2 included STRING interaction detection, immunofluorescence, and Pearson correlation analysis.

**Results:**

ID2 expression was significantly downregulated in MI rats and was inversely correlated with PVC frequency and VT/VF duration. Overexpression of ID2 reduced arrhythmia severity scores and attenuated PVC/VT/VF incidence in MI rats; me’anwhile, it ameliorated mitochondrial dysfunction, as reflected by restored membrane potential and ATP levels and reduced ROS, and improved Na^+^/K^+^-ATPase dysregulation in hypoxia-induced cardiomyocytes. Mechanistically, ID2 directly bound to GATA4 and reduced its expression. Overexpression of ID2 attenuated mitochondrial dysfunction and preserved Na^+^/K^+^-ATPase activity in hypoxic cardiomyocytes, whereas GATA4 overexpression weakened ID2’s protective effect.

**Conclusions:**

This study demonstrates that ID2 alleviates VA by suppressing GATA4 expression, thereby mitigating mitochondrial dysfunction and Na^+^/K^+^-ATPase dysregulation. These findings elucidate the ID2-GATA4 regulatory axis in VA pathogenesis, which may provide a previously unrecognized therapeutic target for arrhythmia management.

## Introduction

Myocardial infarction (MI), a life-threatening cardiovascular condition caused by coronary artery occlusion that leads to myocardial ischemia and necrosis [1], is characterized by high morbidity and mortality rates worldwide [2]. Ventricular arrhythmia (VA), defined as an arrhythmia resulting from abnormal excitation of the myocardium within the ventricular wall, represents a major complication of MI [3]. VA may lead to ventricular tachycardia (VT) or ventricular fibrillation (VF), both of which are closely associated with adverse clinical outcomes [4]. Current pharmacological management of VAs primarily relies on antiarrhythmic agents, including beta-blockers, sodium channel blockers, and calcium channel blockers [5]. However, these therapies remain limited by adverse effects such as dizziness, hypotension, and bradycardia [5], underscoring the urgent need for novel therapeutic strategies targeting the mechanisms of VA. Therefore, investigating the molecular pathways underlying arrhythmogenesis could be important for developing safer and more effective interventions.

Inhibitor of differentiation-2 (ID2), a transcriptional regulator widely expressed in immune cells [6], plays multifunctional roles in tumor growth modulation [7], osteoarthritis amelioration [8], and natural killer cell development [9]. ID2 also acts as an inhibitor of DNA binding by preventing transcription factors from engaging target DNA sequences [10]. Existing research has implicated ID2 in cardiovascular development and disease pathogenesis [11], where it may regulate cardiomyocyte proliferation, inflammatory responses [12], and cytoprotective mechanisms [13]. These findings collectively suggest the potential involvement of ID2 in VA following MI.

GATA4, a transcription factor within the GATA family [14], is indispensable for cardiomyocyte proliferation, embryonic heart development, and cardiac contractile function [15]. Dysregulated GATA4 expression is associated with congenital heart defects, arrhythmias, and heart failure [16], 17]. GATA4 downregulation has also been proposed as a therapeutic strategy for age-related pathological hypertrophy and cardiac dysfunction [14]. Notably, ID2 may serve as an important interaction partner of GATA4, while GATA4 signaling is mechanistically linked to oxidative damage and mitochondrial dysfunction [18], [19], [20]. Based on this evidence, we hypothesize that ID2 may modulate arrhythmogenesis through GATA4-mediated regulation of mitochondrial dysfunction.

The plasma membrane enzyme Na^+^/K^+^-ATPase, which mediates transmembrane Na^+^/K^+^ transport and maintains intracellular ion homeostasis, plays a critical role in regulating vascular tone [21], 22]. Impaired Na^+^/K^+^-ATPase activity contributes to diminished myocardial contractility and is significantly reduced during acute myocardial infarction (AMI) [21], 22]. Notably, the Na^+^/K^+^-ATPase α1/Src signaling axis regulates mitochondrial metabolic signaling in cardiomyocytes [23]. Therefore, aberrant Na^+^/K^+^-ATPase activity is closely associated with post-MI arrhythmias, and elucidating the mechanisms that regulate its activity holds significant pathophysiological importance.

Overall, although previous studies have demonstrated that ID2 plays an important role in cardiac development, its function and underlying mechanisms in post-MI VA remain largely unknown. Moreover, the regulatory relationship between GATA4 and ID2 still lacks direct experimental evidence. Based on these findings, we hypothesize that ID2 may interact with GATA4 to protect mitochondrial function and maintain Na^+^/K^+^-ATPase activity in cardiomyocytes, thereby attenuating VA following MI. To test this hypothesis, we established a rat model of MI via left anterior descending (LAD) coronary artery ligation, combined with a hypoxia-induced cardiomyocyte injury model, to investigate the role of ID2 in post-MI arrhythmia and its potential molecular mechanisms.

## Materials and methods

### Animal grouping and treatment

The study was performed following the ARRIVE guidelines. Animal experiments were approved by the Biomedical Ethics Committee of Jishou University (NO:JSDX-2024-0093). This study was conducted in accordance with the Declaration of Helsinki (as revised in 2013). Male SD rats (SPF grade, 180–200 g), purchased from SJA Laboratory Animal Co., Ltd. (Hunan, China), were acclimatized for one week and subsequently divided into the following groups: Sham, Model, Model + oe-NC, Model + oe-ID2, Model + oe-ID2 + oe-NC, and Model + oe-ID2 + oe-GATA4.

Rats in the Sham and Model groups were anesthetized via intraperitoneal injection of 1 % pentobarbital sodium (50 mg/kg), disinfected, and intubated for oxygen supply. A left thoracotomy was performed by cutting the third rib, followed by pericardial dissection. In the Model group, the LAD coronary artery was permanently ligated with a suture needle inserted approximately 1 cm below the left auricle and passed beneath the vessel, whereas Sham rats underwent identical procedures without ligation before suturing. Subsequently, Model rats received simultaneous multi-point intramyocardial injections in the infarct border zone with adeno-associated virus (AAV, 5 × 10^11^ vg/rat) containing oe-NC, oe-ID2, oe-ID2 + oe-NC, or oe-ID2 + oe-GATA4. Echocardiography was recorded for all groups and for arrhythmia severity.

### Electrocardiograms (ECG) monitoring

ECG monitoring on rats was performed using a non-invasive, wearable telemetry system, as described by Yao et al. [24]. Briefly, after brief anesthesia, epidermal electrodes were attached to the surface of all four limbs of each rat, and the ECG transmitter backpack was then securely fitted onto the rat. After recovery from anesthesia, the rat was allowed to move freely in its cage, during which the system transmitted ECG data in real-time to a mobile terminal. It is important to note that ECG recording was conducted continuously for 24 h to capture premature ventricular contractions (PVCs), ventricular tachycardia (VT), and ventricular fibrillation (VF). On ECG, a PVC was defined as a premature, wide, and aberrant QRS complex, while VT was defined as a run of three or more consecutive, rapid QRS complexes at a rate exceeding 100 beats per minute. VF was identified as chaotic and irregular waveforms without discernible QRS complexes, ST segments, or T-waves [25].

Arrhythmia severity was scored according to the following criteria: Grade 0, ≤49 PVCs; Grade 1, 50–499 PVCs; Grade 2, >499 PVCs and/or one self-terminating episode of VT or VF; Grade 3, one episode of VT or VF lasting <60 s; Grade 4, VT or VF or both lasting 60–119 s; and Grade 5, VT or VF or both lasting ≥120 s [26]. On day 28 post-modeling, after completing all assessments, rats were euthanized via intraperitoneal injection of pentobarbital sodium (150 mg/kg).

### Isolation of neonatal rat cardiomyocytes

Following euthanasia via pentobarbital sodium injection, hearts from neonatal rats were promptly excised and preserved in ice-cold D-Hank’s solution containing 0.25 % trypsin for 12 h at 4 °C. The tissues were then digested with 0.2 % type II collagenase. Isolated cardiomyocytes were resuspended in DMEM (iCell-0001, iCell, China) supplemented with 10 % fetal bovine serum (FBS; 10099141, Gibco, USA) and 1 % penicillin/streptomycin (P/S; SV30010, Beyotime, China), followed by centrifugation. After the cells were allowed to adhere for 1 h, the suspended cells were collected and cultured for 48 h to obtain purified cardiomyocytes.

### Culture and grouping of rat cardiomyocytes

According to the study by Yao et al*.* [26], neonatal rat cardiomyocytes were cultured in DMEM supplemented with 10 % FBS and 1 % P/S at 37 °C in an incubator with 5 % CO_2_. The cells were then divided into the following experimental groups: Control: cells under normal culture conditions; Hypoxia: cells exposed to hypoxia for 24 h in a 37 °C incubator containing 5 % CO_2_, 1 % O_2_, and 94 % N_2_); Hypoxia + oe-NC and Hypoxia + oe-ID2: cells transfected with oe-NC (overexpression-negative control) or oe-ID2 plasmid, respectively, followed by 24 h of hypoxia; Hypoxia + oe-ID2 + DMSO: cells transfected with oe-ID2 plasmid, treated with DMSO (vehicle control) for 24 h, and then exposed to hypoxia for 24 h; Hypoxia + oe-ID2 + Rotenone: cells transfected with oe-ID2 plasmid, treated with 100 nM rotenone for 24 h [27], and then exposed to hypoxia for 24 h; Hypoxia + oe-ID2 + Digoxin: cells transfected with oe-ID2 plasmid, treated with 50 nM digoxin for 24 h [28], and then exposed to hypoxia for 24 h; Hypoxia + si-NC: cells transfected with si-NC (small interfering RNA-negative control), followed by 24 h of hypoxia; Hypoxia + si-GATA4#1 and Hypoxia + si-GATA4#2: cells transfected with si-GATA4#1 or si-GATA4#2, respectively, followed by 24 h of hypoxia; Hypoxia + oe-ID2 + oe-NC: cells co-transfected with oe-ID2 and oe-NC plasmids, followed by 24 h of hypoxia; and Hypoxia + oe-ID2 + oe-GATA4: cells co-transfected with oe-ID2 and oe-GATA4 plasmids, followed by 24 h of hypoxia.

### Immunofluorescence detection of ID2 expression

Myocardial tissue sections from rats were deparaffinized in xylene and a graded ethanol series (100 %, 95 %, 85 %, and 75 %). Antigen retrieval was performed by heating the sections in 0.01 M sodium citrate buffer (pH 6.0), followed by cooling and sequential treatment with sodium borohydride solution, 75 % ethanol, and Sudan Black B staining. After washing, the sections were blocked with 5 % BSA and incubated overnight with the primary ID2 antibody (bs-3515R, Bioss, China). Following PBS washes, the sections were incubated with Goat anti-Rabbit IgG (H + L) Secondary Antibody (AWS0005a, Abiowell, China), counterstained with DAPI (AWC0293a, Abiowell), and mounted with buffered glycerol for fluorescence microscopy observation (BA210T, Motic, China).

Rat cardiomyocytes were fixed with 4 % paraformaldehyde, washed with PBS, and permeabilized with 0.3 % Triton X-100. After PBS rinsing, the cells were blocked with 5 % PBS-BSA and incubated overnight with the primary ID2 antibody. Following PBS washes, the cells were incubated with the secondary antibody, counterstained with DAPI working solution, and mounted with buffered glycerol for fluorescence microscopy observation.

### Detection of ID2 and GATA4 co-localization

Deparaffinized rat myocardial tissue sections were sequentially treated with sodium borohydride solution and 0.3 % hydrogen peroxide, followed by blocking with 5 % BSA. The sections were incubated overnight with the primary ID2 antibody, washed with PBS, and then incubated with the secondary antibody. After PBS washes, the sections were treated with TYP-520 fluorescent dye (AWI0693, Abiowell), rinsed, and subjected to antigen retrieval by heating in 0.01 M sodium citrate buffer (pH 6.0). After cooling, the sections were washed with PBS, re-blocked with 0.3 % hydrogen peroxide and 5 % BSA, and incubated overnight with the primary GATA4 antibody (bs-23982R, Bioss, China). Following PBS washes, an anti-rabbit IgG secondary antibody was applied. After final PBS rinsing, the sections were counterstained with TYP-620 fluorescent dye and DAPI working solution, and then mounted with buffered glycerol for fluorescence microscopy analysis.

### Western blot

Proteins were extracted from rat myocardial tissues and cardiomyocytes using RIPA lysis buffer (AWB0136, Abiowell), and protein concentrations were quantified using a BCA assay kit (AWB0104, Abiowell). Denatured proteins were separated by gel electrophoresis and transferred onto nitrocellulose (NC) membranes. The membranes were blocked with 5 % skim milk in 1 × PBST (PBS with 0.1 % Tween-20) and incubated overnight with primary antibodies. After washing with 1 × PBST, the membranes were incubated with species-matched secondary antibodies. Protein bands were visualized using an ECL chemiluminescent substrate and imaged with a gel documentation system (ChemiScope6100, Clinx Science, China). The antibody details are provided in Table 1. The full-length blots are included in Supplementary File S1.

**Table 1:** The information on the antibody.

Name	Dilution rate	Cat. number	Source	Company	Country
ID2	1:500	ab90055	Mouse	Abcam	UK
Nav1.2, SCN2A	1:1,000	A10574	Rabbit	ABclonal	China
KCNQ3	1:600	19966-1-AP	Rabbit	Proreintech	USA
Cav1.2	1:1,000	ab84814	Mouse	Abcam	UK
P-troponin I	1:1,000	ab58546	Rabbit	Abcam	UK
Troponin I	1:2,500	ab209809	Rabbit	Abcam	UK
Calmodulin	1:1,000	10541-1-AP	Rabbit	Proreintech	USA
GATA4	1:3,000	19530-1-AP	Rabbit	Proreintech	USA
GAPDH	1:5,000	10494-1-AP	Rabbit	Proreintech	USA
HRP goat anti- mouse IgG (H + L)	1:5,000	SA00001-1	Goat	Proteintech	USA
HRP goat anti- rabbit IgG (H + L)	1:6,000	SA00001-2	Goat	Proteintech	USA

### Real-time quantitative polymerase chain reaction (RT-qPCR)

Total RNA was extracted from rat myocardial tissues and cells using TRIzol reagent (15596026, Thermo, USA). cDNA was synthesized using the mRNA Reverse Transcription Kit (CW2569, CwBio, China). Reaction mixtures containing cDNA, ID2-and GATA4-specific primers, 2 × SYBR PCR Master Mix, and ddH_2_O (CW2601, CwBio) were prepared and amplified on a real-time PCR system (SPL0960, Thermo). GAPDH served as the endogenous control. Primer sequences are detailed in Table 2.

**Table 2:** Primer sequences.

Gene	Sequence (5′-3′)	Length
R-ID2	F ACGACTGCTACTCCAAGCTC R CAGGATGCTGATGTCCGTGT	221 bp
R-GATA4	F GCC​AAC​TGC​CAG​ACT​ACC​AC	162 bp
R-GAPDH	F ACAGCAACAGGGTGGTGGAC R TTTGAGGGTGCAGCGAACTT	252 bp

### Analysis of mitochondrial membrane potential

Following the instructions of the JC-1 Assay Kit (C2006, Beyotime), a staining working solution was prepared and added to the cells resuspended in culture medium. After incubation for 20 min in a cell culture incubator, the cells were centrifuged, and the pellet was retained. The cells were washed twice with 1 × JC-1 staining buffer, with centrifugation and supernatant removal after each wash, and were finally resuspended in fresh 1 × JC-1 staining buffer for analysis using a flow cytometer (A00-1-110, Beckman, USA).

### Detection of ROS levels

Following the protocol of the Mito-Tracker Green Kit (C1048, Beyotime), the stock solution (1 mM) and working solution (200 nM) were prepared. After the cell culture medium was aspirated, pre-warmed working solution (37 °C) was added to the cells, followed by incubation for 30 min. The working solution was then removed and replaced with fresh culture medium, and ROS levels were analyzed using a flow cytometer.

### ATP content assay

Myocardial tissue or cell pellets were homogenized on ice with cold double-distilled water at a ratio of 1:9 to obtain homogenates. After protein concentration determination, the tissue or cell homogenates were heated in a boiling water bath for 10 min. The homogenates were then centrifuged to collect the supernatants. Subsequently, ATP content in the supernatants was measured according to the instructions of the commercial assay kit (A095-1-1, njjcbio, China). Finally, the absorbance of the test samples was measured at a wavelength of 636 nm.

### Na^+^/K^+^-ATPase activity assay

Myocardial tissue was homogenized on ice with normal saline, and the supernatant of the homogenate was collected and diluted with normal saline to a 1 % concentration. Meanwhile, a suspension of cardiomyocytes was collected and subjected to ultrasonic disruption. Subsequently, Na^+^/K^+^-ATPase activity was measured according to the instructions of the commercial assay kit (Kit No. BC0515, njjcbio).

### Mitochondrial respiratory chain complex activity assay

Extraction buffer was added to myocardial tissues or cells, followed by homogenization using an ice-bath homogenizer. The homogenate was centrifuged at 600×*g* at 4 °C, and the supernatant was collected. This supernatant was then centrifuged at 11,000×*g*, and the resulting pellet was retained. According to the respective instructions of the commercial assay kits for NADH dehydrogenase (Complex I, BC0515, njjcbioa), succinate dehydrogenase (Complex II, BC3235, njjcbio), cytochrome *c* oxidase (Complex IV, BC0945, njjcbio), and ATP synthase (Complex V, BC1445, njjcbio), the corresponding reagents were added to the pellet and mixed thoroughly. The mixed samples were then subjected to ultrasonic disruption. Subsequently, the optical density (OD) values were determined using a microplate reader (MB-530, HEALES, China) at 340 nm for Complex I, 605 nm for Complex II, 550 nm for Complex IV, and 606 nm for Complex V.

### Statistical analysis

**Figure 1:**
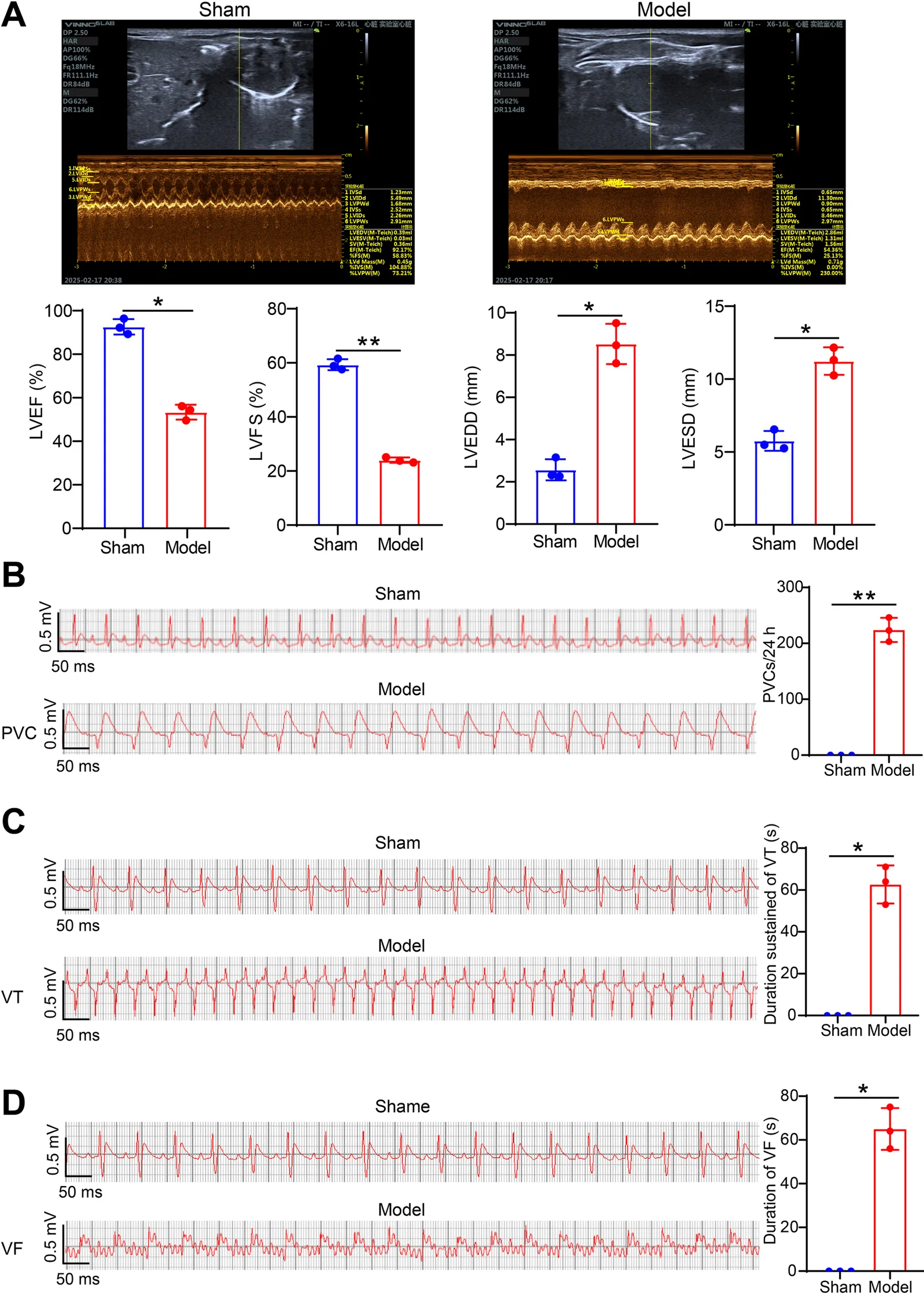

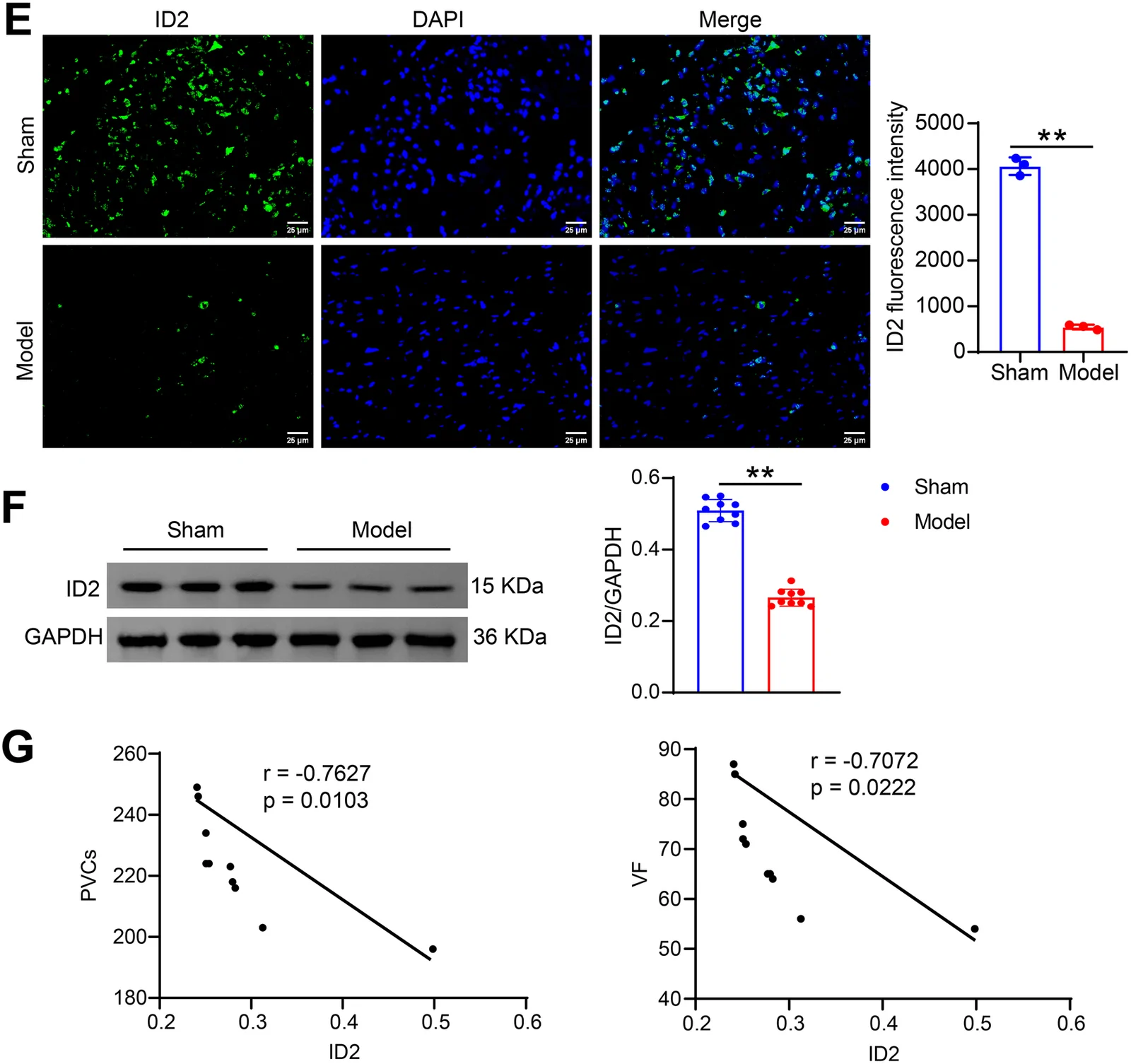
LAD-induced MI rats exhibit arrhythmias and downregulate ID2 expression. (A) Echocardiographic analysis of LVEF, LVFS, LVEDD, and LVESD in rats. (B–D) ECG recordings and statistical analysis of PVCs, VT, and VF duration in rats. (E) Immunofluorescence detection of ID2 expression in myocardial tissue (magnification: ×400, scale bar=25 μm). (F) Western blot detection of ID2 expression in myocardial tissues. (G) Pearson correlation analysis of ID2 expression with PVCs and VF duration. Data are represented as the meanᵒ±ᵒSD, n=3 rats per group, representing biological replicates. Statistical significance was determined using Student’s t-test. *p<0.05, **p<0.01. r<0 indicates negative correlation.

**Figure 2:**
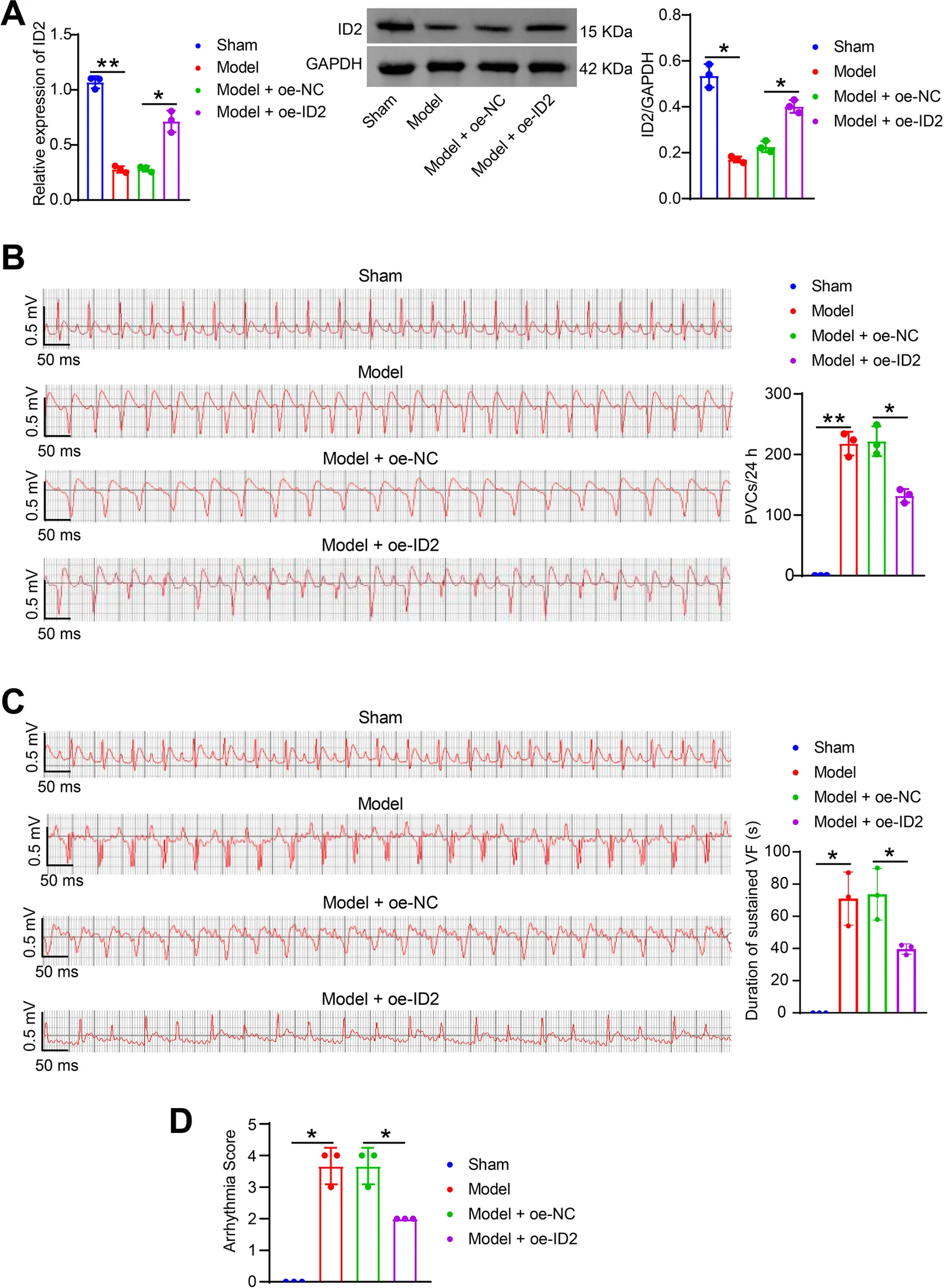
ID2 attenuates arrhythmia incidence in LAD-induced MI rats by reducing PVCs, VT, and VF. (A) RT-qPCR and western blot detection of ID2 expression in myocardial tissue. (B and C) ECG recordings and statistical analysis of PVCs and VF duration in rats. (D) Arrhythmia scoring in rats. Data are represented as the meanᵒ±ᵒSD, n=3 rats per group, representing biological replicates. Statistical significance was determined using one-way ANOVA with Tukey’s multiple comparisons test. *p<0.05, **p<0.01.

The data were analyzed using GraphPad Prism 9.0 and are presented as the meanᵒ±ᵒstandard deviation (SD). Data distribution was first assessed using the Kolmogorov-Smirnov test and exploratory descriptive statistics. Data meeting the assumptions of normal distribution and homogeneity of variance were included in subsequent analyses. Comparisons between two groups were performed using the unpaired Student’s t-test, as shown in Figure 1A–F. Differences among multiple groups were assessed by one-way analysis of variance (ANOVA) with Tukey’s post-hoc test, as applied to the analysis of ID2 expression in myocardial tissue presented in Figure 2A. Pearson correlation coefficients (r) were used to quantify the expression relationship between ID2 and GATA4, with r<0 indicating a negative correlation. A p<0.05 was considered statistically significant.

## Results

### LAD-induced MI rats exhibit arrhythmias and downregulate ID2 expression

A rat MI model was successfully established via LAD ligation. Echocardiographic analysis revealed that rats in the Model group exhibited significantly reduced left ventricular ejection fraction (LVEF) and fractional shortening (LVFS) compared with the Sham group, accompanied by increased left ventricular end-systolic diameter (LVESD) and left ventricular end-diastolic diameter (LVEDD) (Figure 1A). Electrocardiographic recordings demonstrated frequent VA in Model rats, including PVCs (>200 events/24 h), VT, and VF, with VT/VF episodes lasting 60 s. These findings confirmed the successful induction of arrhythmias secondary to MI (Figure 1B–D). Notably, ID2 expression was significantly downregulated in the myocardial border zone of rats in the Model group (Figure 1E and F), suggesting that ID2 loss may be associated with post-MI myocardial injury. Pearson correlation analysis further revealed a negative association between ID2 levels and arrhythmia severity, including PVC frequency and VF duration, suggesting that aberrant ID2 expression may contribute to arrhythmogenesis in LAD-induced MI (Figure 1G).

### ID2 attenuates arrhythmia incidence in LAD-induced MI rats by reducing PVCs, VT, and VF

To investigate the role of ID2 in MI-associated arrhythmias, we observed that ID2 expression was significantly downregulated in rats in the Model group, whereas administration of AAV containing oe-ID2 restored its expression (Figure 2A). Concomitantly, ID2 upregulation markedly reduced arrhythmia severity, as shown by decreased PVC frequency and shortened VF duration in MI rats (Figure 2B and C). Arrhythmia severity scoring further confirmed that ID2 overexpression lowered arrhythmia scores compared with the Model group (Figure 2D). These results collectively demonstrate that ID2 ameliorates arrhythmogenesis in LAD-induced MI rats.

### Overexpression of ID2 attenuates mitochondrial dysfunction and Na^+^/K^+^-ATPase activity dysregulation in MI rats

**Figure 3:**
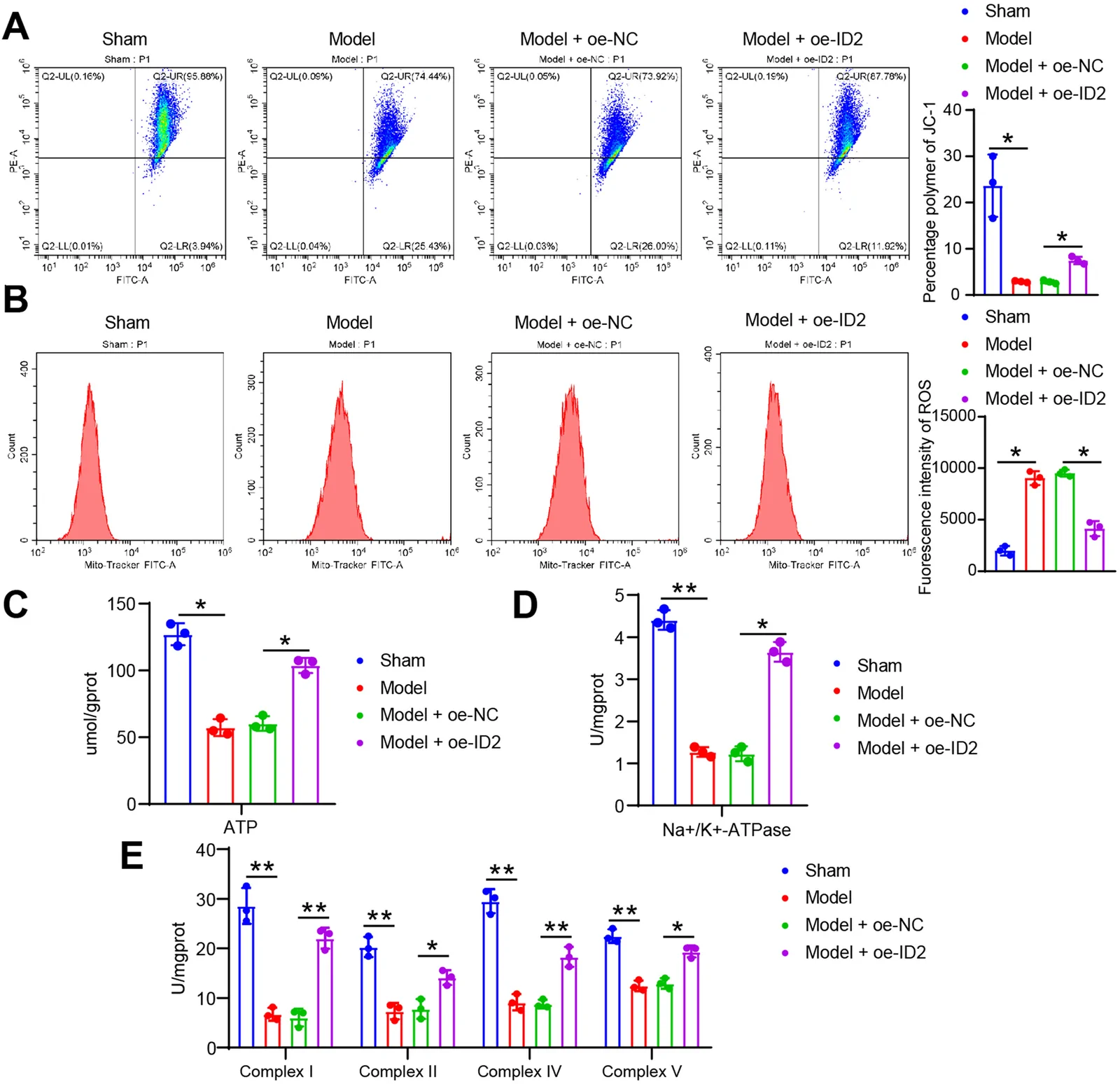
Overexpression of ID2 attenuates mitochondrial dysfunction and Na^+^/K^+^-ATPase activity dysregulation in MI rats. (A) Detection of mitochondrial membrane potential (B) Detection of ROS in mitochondria. (C) Detection of ATP synthesis. (D) Na^+^/K^+^-ATPase activity assay. (E) Detection of Complex I, Complex II, Complex IV, and Complex V levels. Data are represented as the meanᵒ±ᵒSD, n=3 rats per group, representing biological replicates. Statistical significance was determined using one-way ANOVA with Tukey’s multiple comparisons test. *p<0.05, **p<0.01.

Post-MI VA is known to correlate with oxidative damage and mitochondrial dysfunction [29]. To further elucidate the mechanism by which ID2 mitigates arrhythmias in MI rats, we investigated its role in mitochondrial function. Our data revealed that rats in the Model group exhibited reduced mitochondrial membrane potential, diminished ATP levels, and elevated mitochondrial ROS levels, indicating impaired mitochondrial function. ID2 overexpression restored mitochondrial membrane potential, increased ATP production, and lowered ROS levels (Figure 3A–C). Moreover, compared with the Sham group, Model rats showed decreased activities of mitochondrial respiratory chain complexes (Complexes I, II, IV, and V), together with reduced Na^+^/K^+^-ATPase activity. These deficits were reversed by ID2 overexpression (Figure 3D and E). Collectively, these findings demonstrate that ID2 may alleviate arrhythmias in MI rats by suppressing mitochondrial dysfunction and restoring Na^+^/K^+^-ATPase activity.

### Overexpression of ID2 attenuates hypoxia-induced mitochondrial dysfunction and Na^+^/K^+^-ATPase dysregulation in cardiomyocytes

**Figure 4:**
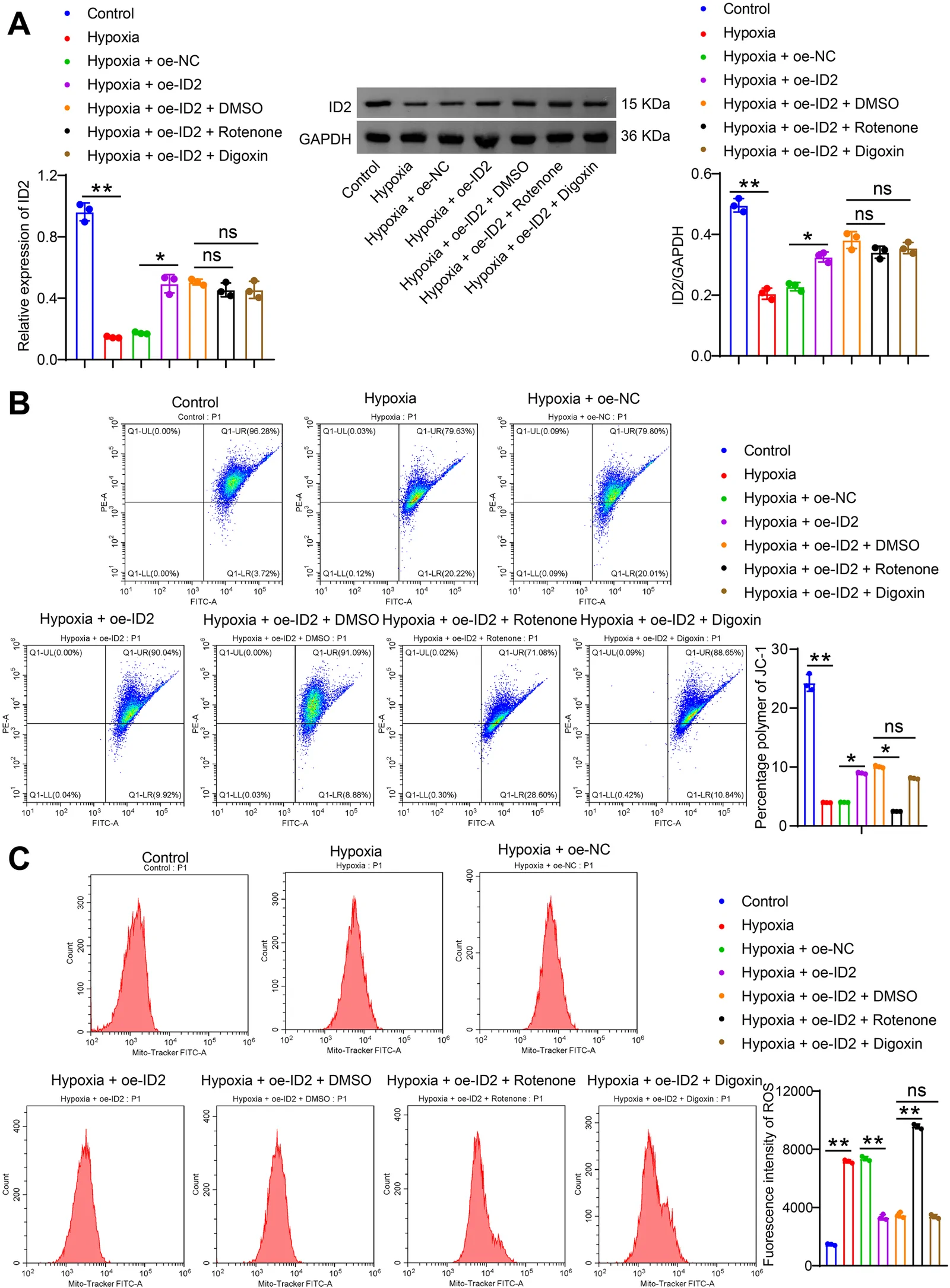

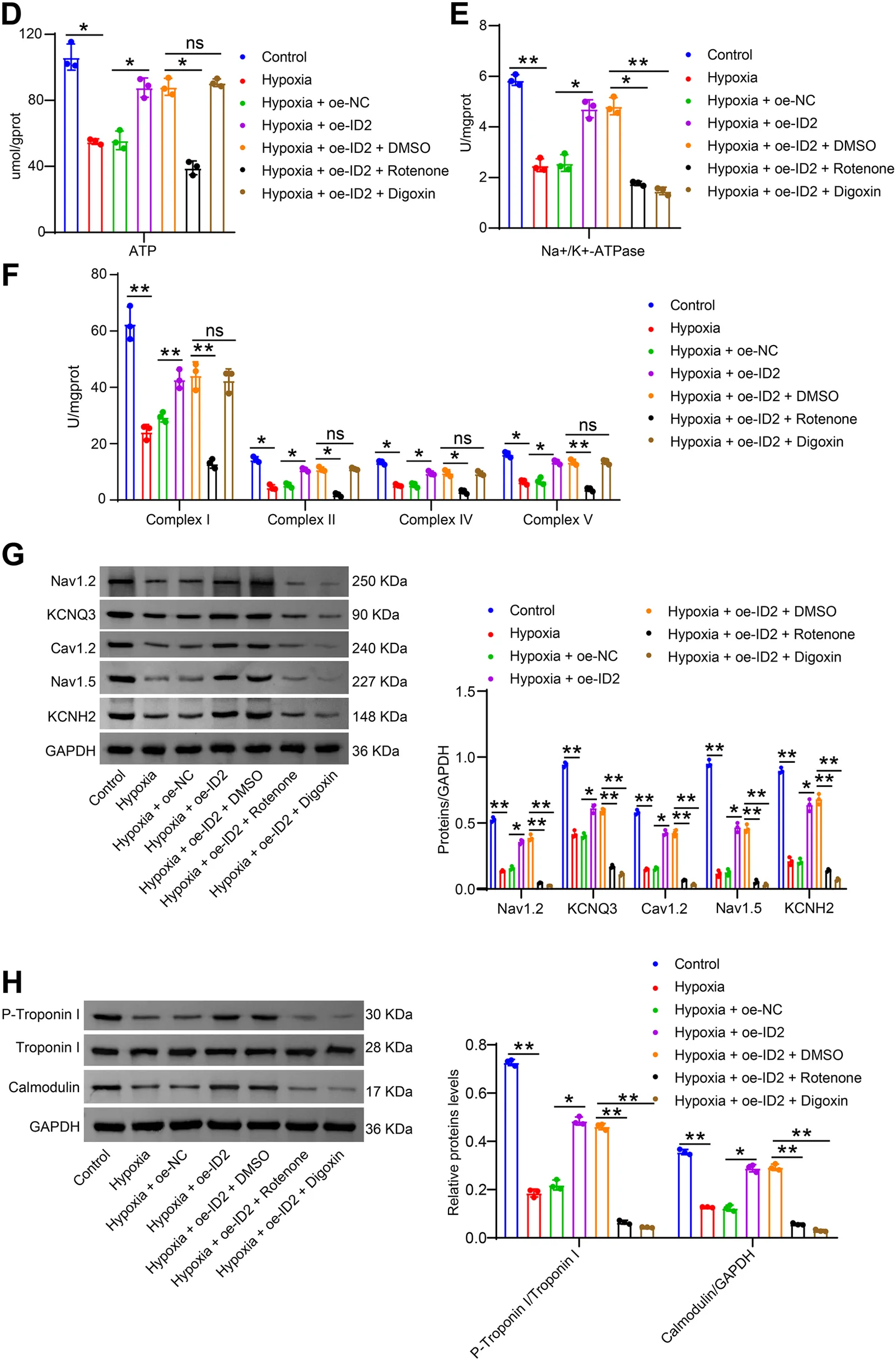
Overexpression of ID2 attenuates hypoxia-induced mitochondrial dysfunction and Na^+^/K^+^-ATPase dysregulation in cardiomyocytes. (A) RT-qPCR and western blot detection of ID2 expression in hypoxic cardiomyocytes. (B) Detection of mitochondrial membrane potential. (C) Detection of mitochondrial ROS levels. (D) Detection of ATP synthesis. (E) Na^+^/K^+^-ATPase activity assay. (F) Detection of Complex I, Complex II, Complex IV, and Complex V activities. (G-H) Western blot assay for the expression levels of ion channel proteins (Nav1.2, Nav1.5, KCNH2, KCNQ3, and Cav1.2), troponins (Troponin I and p-Troponin I), and the calmodulin protein Calmodulin. Data are represented as the meanᵒ±ᵒSD, n=3 biological replicates. Statistical significance was determined using one-way ANOVA with Tukey’s multiple comparisons test. *ns* indicates no significance; *p<0.05, **p<0.01.

Next, the mechanism by which ID2 mitigates myocardial injury through mitochondrial regulation was further investigated *in vitro*. Primary cardiomyocytes were first isolated from neonatal rats. Figure S1A shows representative images of the isolated neonatal rat cardiomyocytes immediately after isolation and after 24 h in culture. The images demonstrate that the isolated cardiomyocytes exhibited intact cellular structure, normal morphology, appropriate density, and good adherence (Figure S1B). Subsequently, the cardiomyocytes were transfected with the oe-ID2 plasmid, which increased ID2 expression and confirmed the successful manipulation of ID2 (Figure S1A). Following this, a hypoxia model was established in rat cardiomyocytes, which were then treated with the Complex I inhibitor rotenone or the Na^+^/K^+^-ATPase inhibitor digoxin. As shown in Figure 4A, ID2 expression was downregulated in the hypoxia group compared with the control group, while this reduction was restored by transfection with the oe-ID2 plasmid. Rotenone and digoxin treatment did not significantly alter ID2 expression (Figure 4A). Furthermore, Figure S1C–E show that the cardiomyocytes used for the detection of mitochondrial membrane potential, ROS, and flow cytometry were in good condition. We observed that ID2 overexpression reversed the hypoxia-induced reductions in mitochondrial membrane potential and ATP levels, while also attenuating ROS elevation, demonstrating its protective effect against hypoxia-induced mitochondrial impairment (Figure 4B–D). Notably, rotenone treatment in the Hypoxia + oe-ID2 + Rotenone group weakened the inhibitory effect of ID2 overexpression on mitochondrial dysfunction, as evidenced by reduced membrane potential, decreased ATP levels, and elevated ROS levels, whereas digoxin treatment in the Hypoxia + oe-ID2 + Digoxin group showed no significant impact on these mitochondrial parameters (Figure 4B–D). In addition, hypoxia suppressed the activities of Complexes I, II, IV, and V, as well as Na^+^/K^+^-ATPase activity, while ID2 overexpression reversed these deficits and restored enzymatic activities. However, rotenone and digoxin counteracted the protective effects of ID2 overexpression, thereby exacerbating mitochondrial dysfunction and Na^+^/K^+^-ATPase dysregulation (Figure 4E and F). In addition, Figure 4G and H revealed that ID2 overexpression upregulated the levels of ion channel proteins (Nav1.2, Nav1.5, KCNH2, KCNQ3, and Cav1.2), troponin-related proteins (p-Troponin I and Troponin I), and calmodulin. Rotenone and digoxin treatment reduced these protein levels, thereby weakening the benefits of ID2 overexpression. Collectively, these findings demonstrate that ID2 overexpression could alleviate hypoxia-induced mitochondrial dysfunction and Na^+^/K^+^-ATPase dysregulation in cardiomyocytes.

### Overexpression of ID2 suppresses GATA4 protein expression

**Figure 5:**
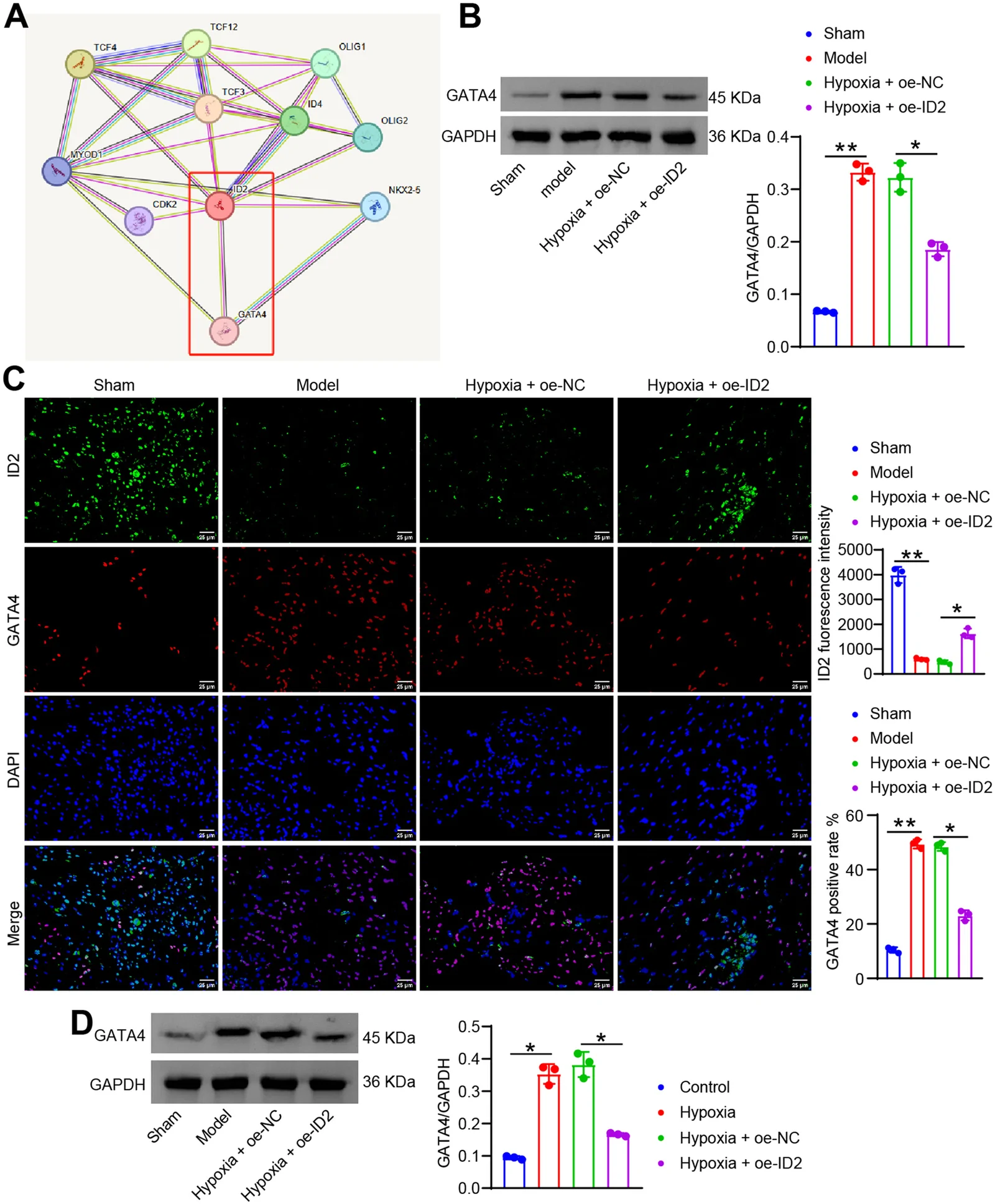

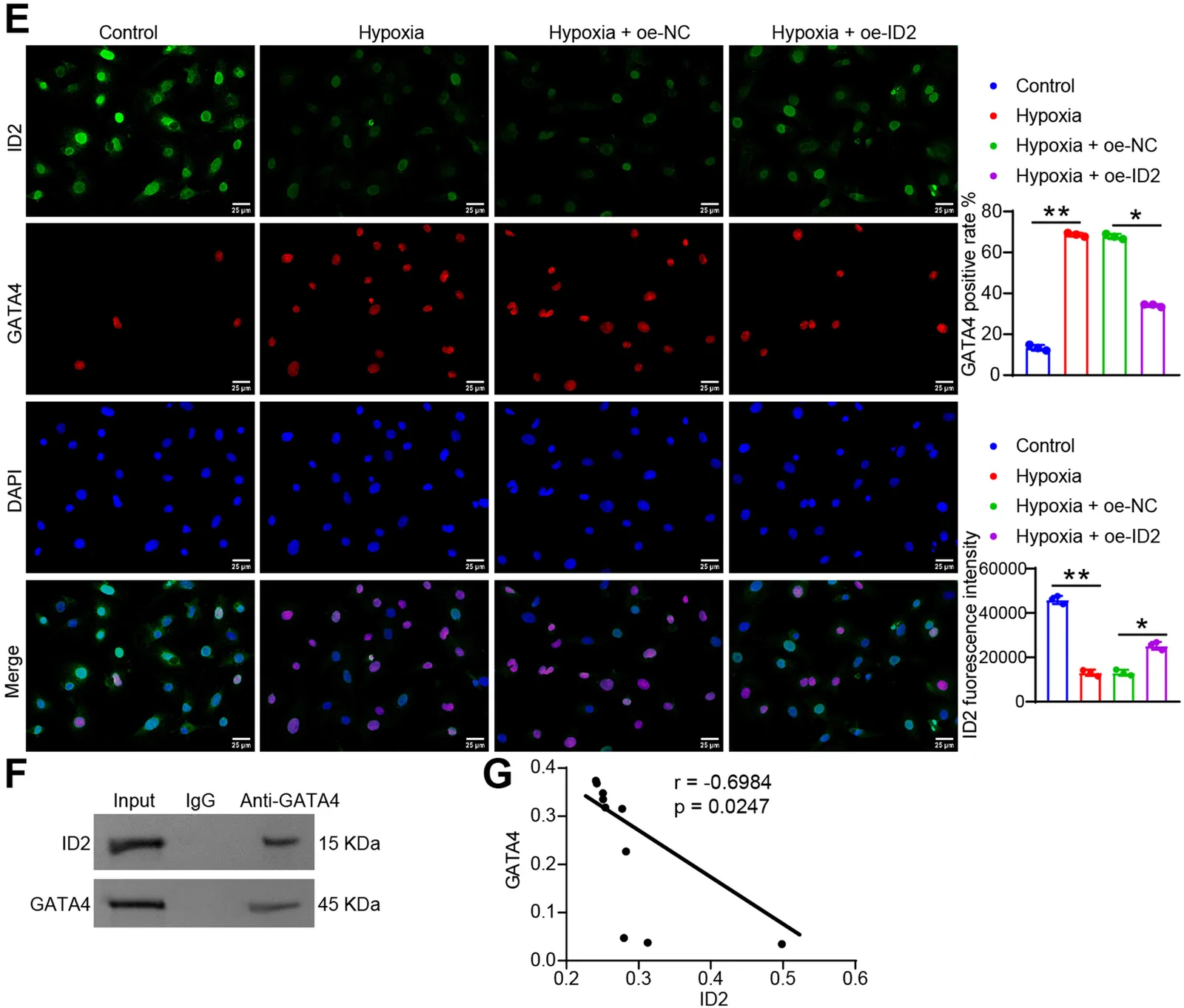
Overexpression of ID2 suppresses GATA4 protein expression. (A) STRING analysis of the interaction between ID2 and GATA4. (B) Western blot detection of GATA4 expression in myocardial tissues. (C) Immunofluorescence detection of the co-localization of ID2 and GATA4 in myocardial tissues (magnification: ×400, scale bar=25 μm). (D) Western blot detection of GATA4 expression in cardiomyocytes. (E) Immunofluorescence detection of the co-localization of ID2 and GATA4 in cardiomyocytes (magnification: ×400, scale bar=25 μm). (F) Co-IP detection of the interaction between ID2 and GATA4. (G) Pearson correlation analysis between ID2 and GATA4. Data are represented as the meanᵒ±ᵒSD, n=3 biological replicates. Statistical significance was determined using one-way ANOVA with Tukey’s multiple comparisons test. *p<0.05, **p<0.01. r<0 indicates negative correlation.

We further investigated the mechanism by which ID2 modulates mitochondrial dysfunction. Previous literature suggests that GATA4 may regulate sodium channel genes implicated in arrhythmogenesis [30], while STRING database analysis revealed a potential interaction between ID2 and GATA4 (Figure 5A). In the Model group, GATA4 expression was abnormally increased, whereas this increase was suppressed upon ID2 overexpression (Figure 5B). ID2 and GATA4 were co-localized in myocardial tissues, and GATA4 protein levels decreased with ID2 upregulation (Figure 5C). These findings were further corroborated in the *in vitro* hypoxia model, as evidenced by the co-localization of ID2 and GATA4 in cardiomyocytes and the concomitant downregulation of GATA4 expression following ID2 overexpression (Figure 5D and E). Furthermore, Co-IP confirmed a direct interaction between ID2 and GATA4, with a negative correlation observed between their expression levels (Figure 5F and G). These results suggest that ID2 negatively regulates GATA4 expression, potentially positioning GATA4 as a downstream target of ID2.

### GATA4 knockdown alleviates mitochondrial dysfunction and Na^+^/K^+^-ATPase dysregulation in hypoxic cardiomyocytes

**Figure 6:**
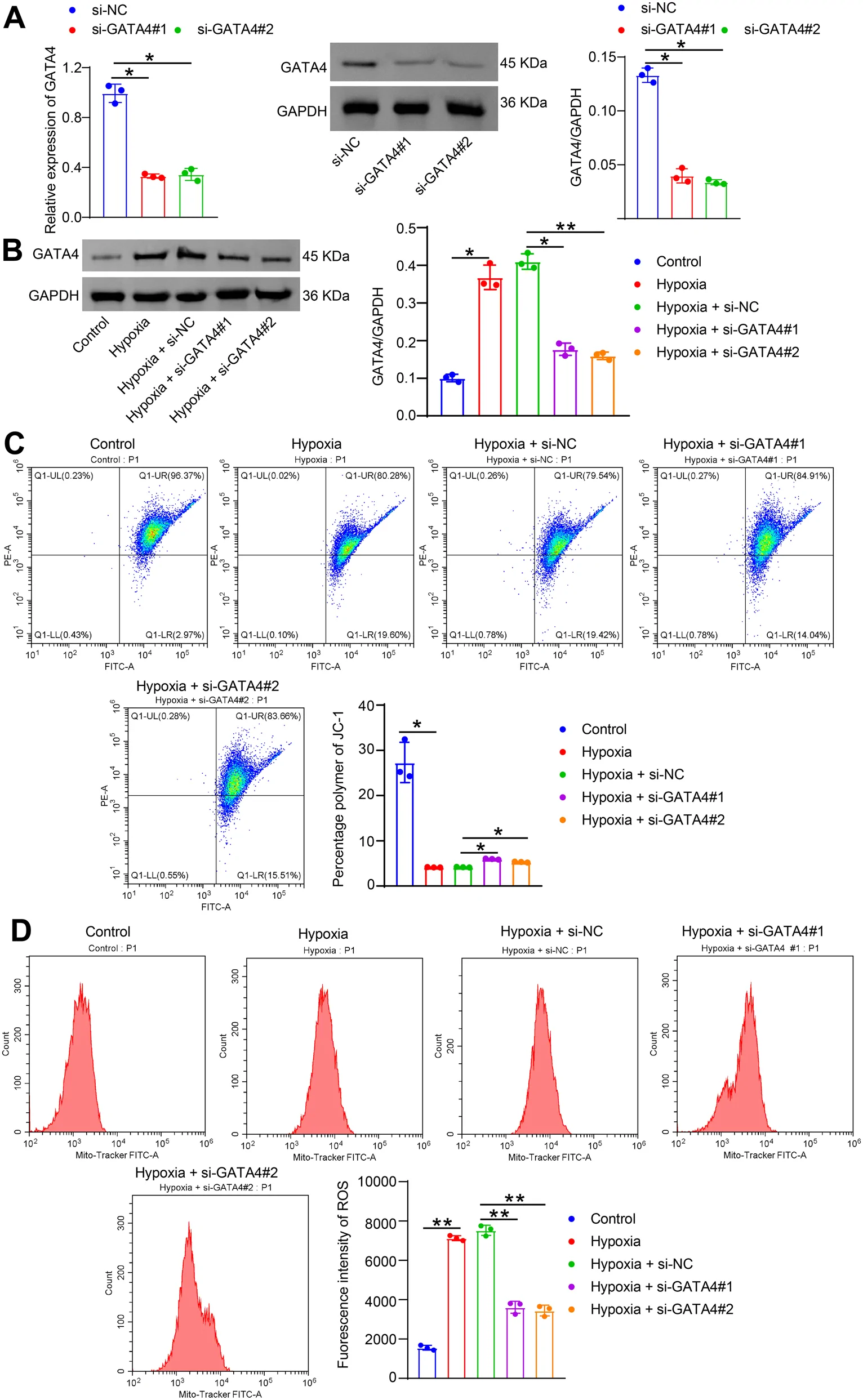

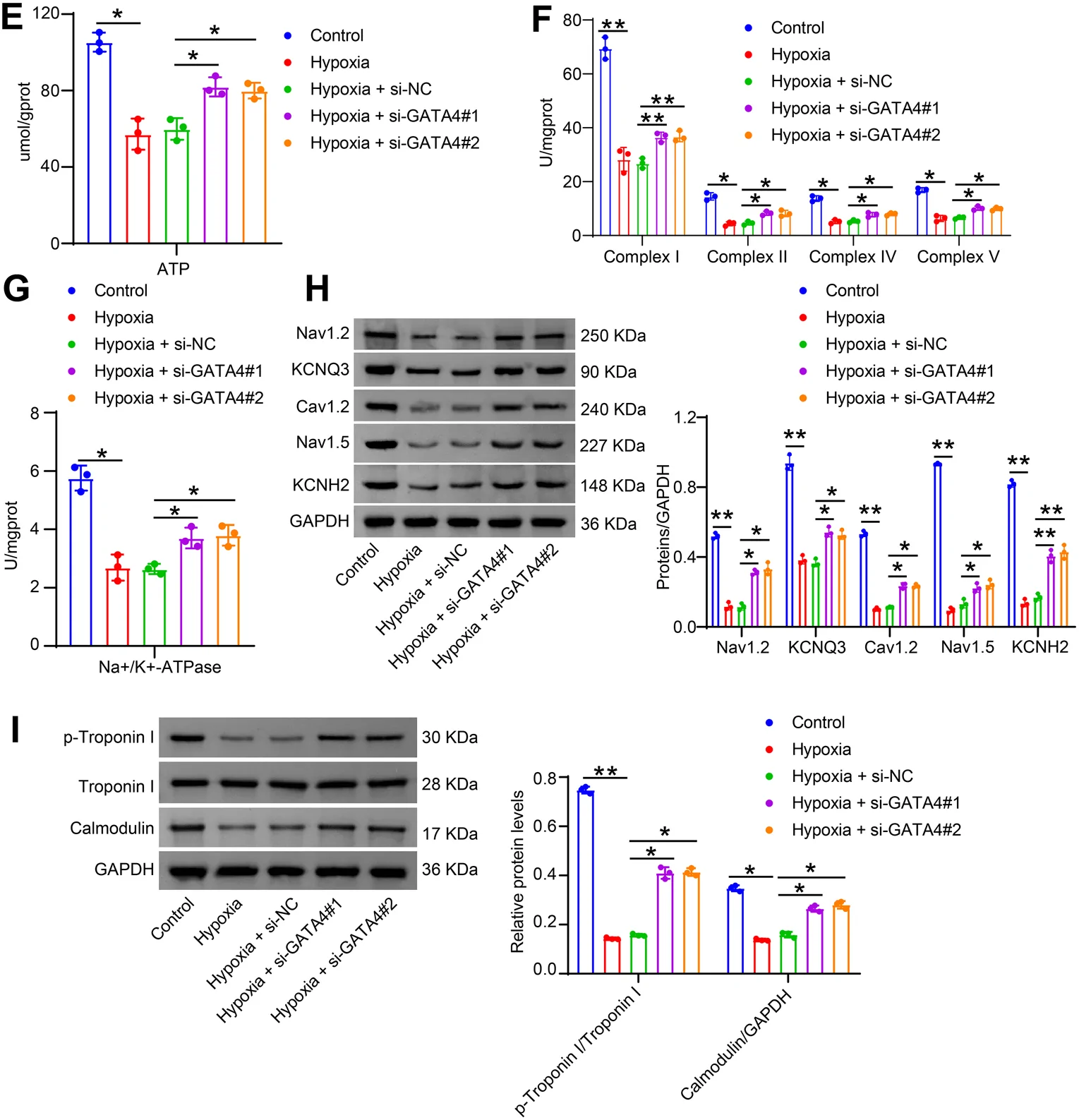
GATA4 knockdown alleviates mitochondrial dysfunction and Na^+^/K^+^-ATPase dysregulation in hypoxic cardiomyocytes. (A) RT-qPCR and western blot detection of GATA4 transfection efficiency in cardiomyocytes. (B) Western blot detection of GATA4 expression in hypoxic cardiomyocytes. (C) Detection of mitochondrial membrane potential. (D) Detection of mitochondrial ROS levels. (E) Detection of ATP synthesis. (F) Detection of Complex I, Complex II, Complex IV, and Complex V activities. (G) Na^+^/K^+^-ATPase activity assay. (H-I) Western blot assay for the expression levels of ion channel proteins (Nav1.2, Nav1.5, KCNH2, KCNQ3, and Cav1.2), troponins (Troponin I and p-Troponin I), and the calmodulin protein Calmodulin. Data are represented as the meanᵒ±ᵒSD, n=3 biological replicates. Statistical significance was determined using one-way ANOVA with Tukey’s multiple comparisons test. *p<0.05, **p<0.01.

To investigate whether GATA4 influences mitochondrial function in cardiomyocytes, GATA4 knockdown plasmids were transfected into cells, which significantly reduced GATA4 expression (Figure 6A). Hypoxia treatment increased GATA4 levels in cardiomyocytes, whereas this increase was decreased by GATA4 knockdown (Figure 6B). Intriguingly, GATA4 silencing improved mitochondrial function, as reflected by increased mitochondrial membrane potential and ATP levels, together with decreased ROS levels (Figure 6C–E). Concurrently, the activities of Complexes I, II, IV, and V, as well as Na^+^/K^+^-ATPase activity, were elevated in GATA4-knockdown cells under hypoxia (Figure 6F and G). Additionally, GATA4 knockdown upregulated the expression of Nav1.2, Nav1.5, KCNH2, KCNQ3, Cav1.2, p-Troponin I/Troponin I, and calmodulin in hypoxic cardiomyocytes (Figure 6H and I). These findings suggest that GATA4 knockdown alleviates hypoxia-induced mitochondrial dysfunction and Na^+^/K^+^-ATPase dysregulation.

### ID2 alleviates hypoxia-induced mitochondrial dysfunction and Na^+^/K^+^-ATPase dysregulation in cardiomyocytes via GATA4 downregulation

**Figure 7:**
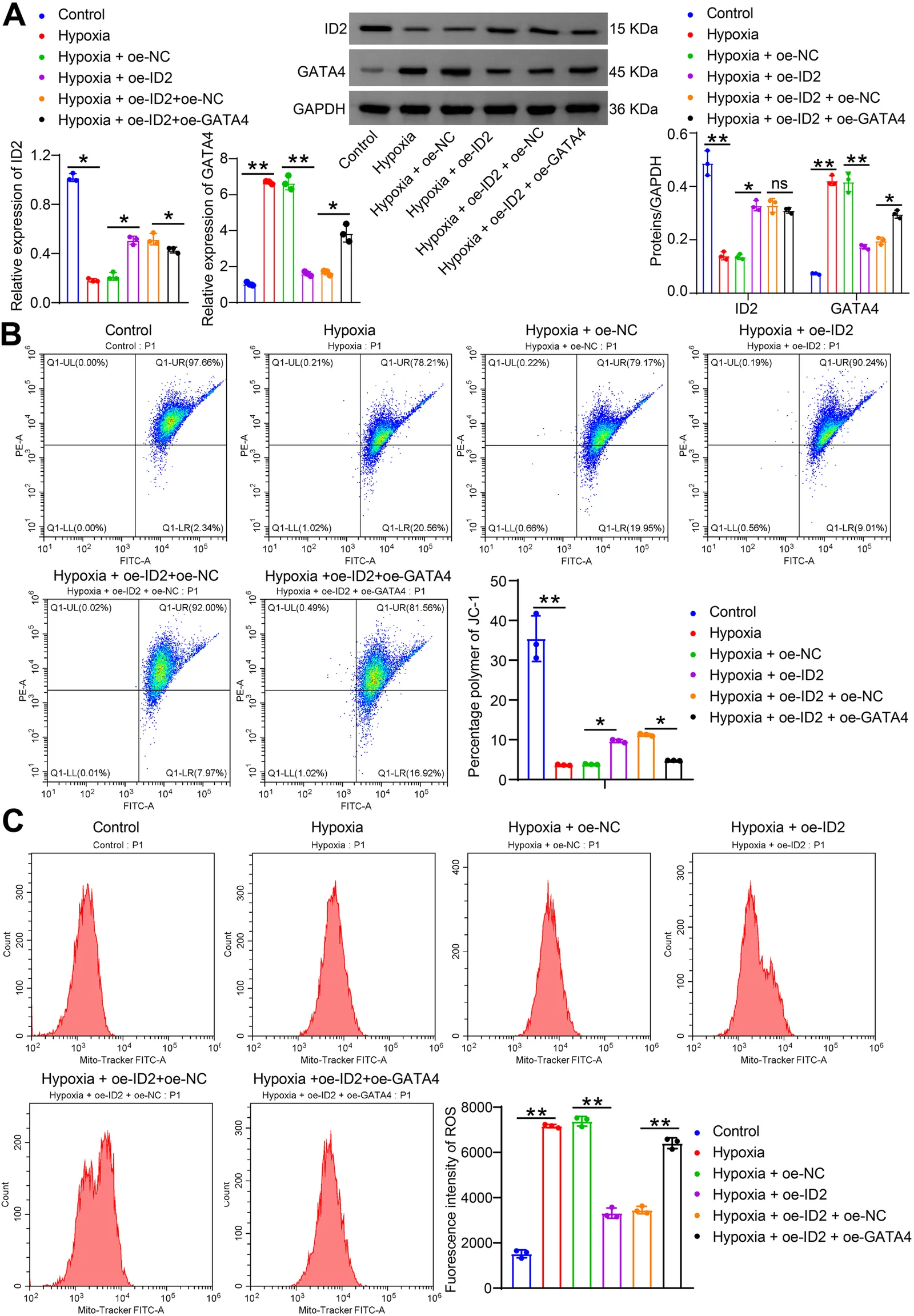

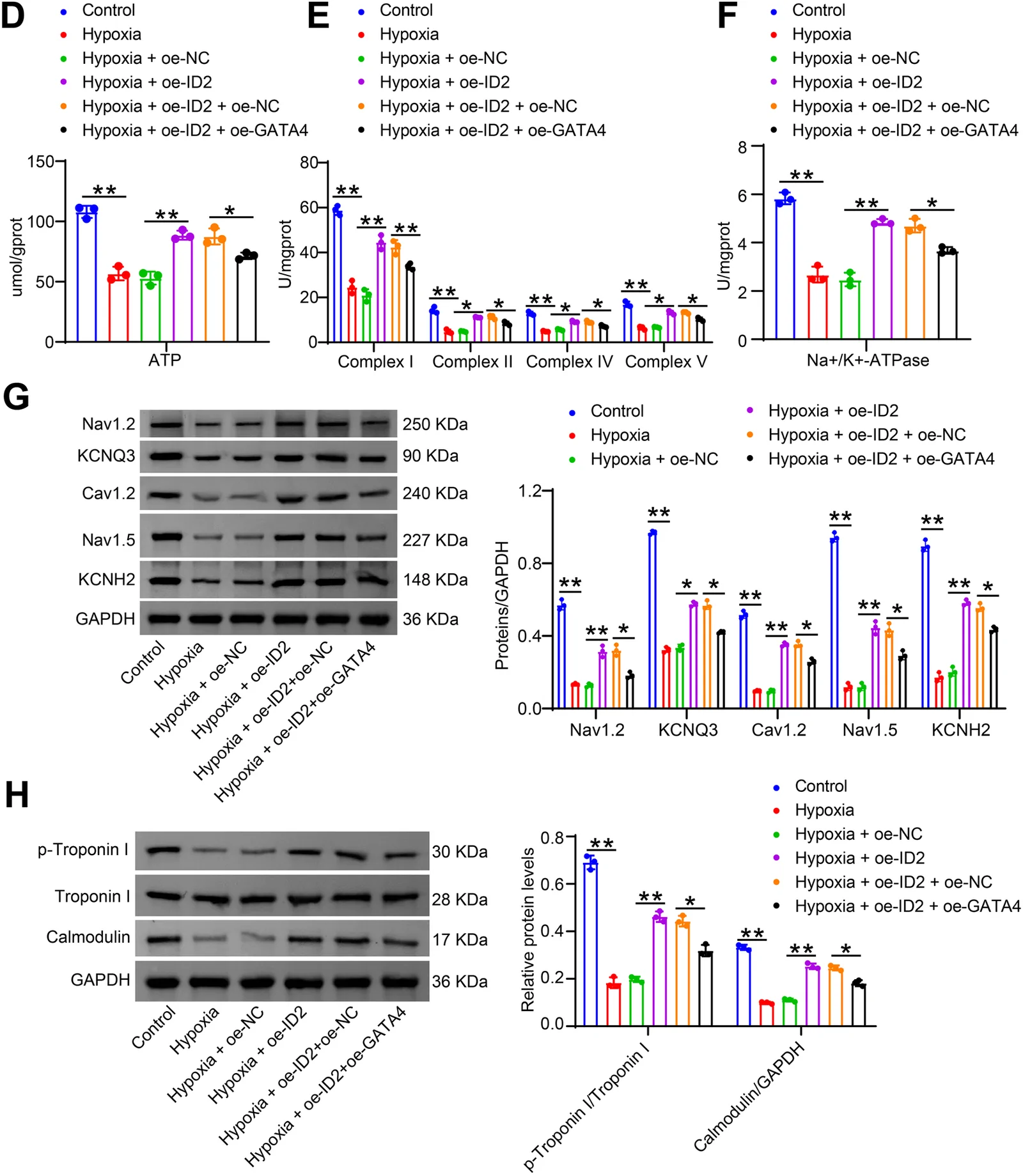
ID2 alleviates hypoxia-induced mitochondrial dysfunction and Na^+^/K^+^-ATPase dysregulation in cardiomyocytes via GATA4 downregulation. (A) RT-qPCR and western blot detection of ID2 and GATA4 expression in hypoxic cardiomyocytes. (B) Detection of mitochondrial membrane potential. (C) Detection of mitochondrial ROS levels. (D) Detection of ATP synthesis. (E) Detection of Complex I, Complex II, Complex IV, and Complex V activities. (F) Na^+^/K^+^-ATPase activity assay. (G-H) Western blot assay for the expression levels of ion channel proteins (Nav1.2, Nav1.5, KCNH2, KCNQ3, and Cav1.2), troponins (Troponin I and p-Troponin I), and the calmodulin protein Calmodulin. Data are represented as the meanᵒ±ᵒSD, n=3 biological replicates. Statistical significance was determined using one-way ANOVA with Tukey’s multiple comparisons test. *ns* indicates no significance; *p<0.05, **p<0.01.

To further elucidate the regulatory mechanism of ID2 on GATA4, hypoxic cardiomyocytes were co-transfected with ID2 and GATA4 overexpression plasmids. Compared with the Hypoxiaᵒ+ᵒoe-NC group, ID2 expression was increased, while GATA4 expression was decreased in the Hypoxiaᵒ+ᵒoe-ID2 group. In contrast, GATA4 overexpression elevated GATA4 levels without significantly altering ID2 expression (Figure 7A). Mitochondrial functional assays revealed that GATA4 overexpression reversed the protective effects of ID2 by reducing mitochondrial membrane potential and ATP levels while increasing ROS accumulation (Figure 7B–D). Furthermore, GATA4 overexpression suppressed the activities of Complexes I, II, IV, and V, as well as Na^+^/K^+^-ATPase activity, while downregulating the expression of Nav1.2, Nav1.5, KCNH2, KCNQ3, Cav1.2, p-Troponin I/Troponin I, and calmodulin, thereby reversing the therapeutic benefits of ID2 overexpression (Figure 7E–H). These findings demonstrate that ID2 mitigates hypoxia-induced mitochondrial dysfunction and Na^+^/K^+^-ATPase dysregulation by suppressing GATA4 expression.

### Overexpression of ID2 attenuates LAD-induced arrhythmias in MI rats via GATA4 downregulation

**Figure 8:**
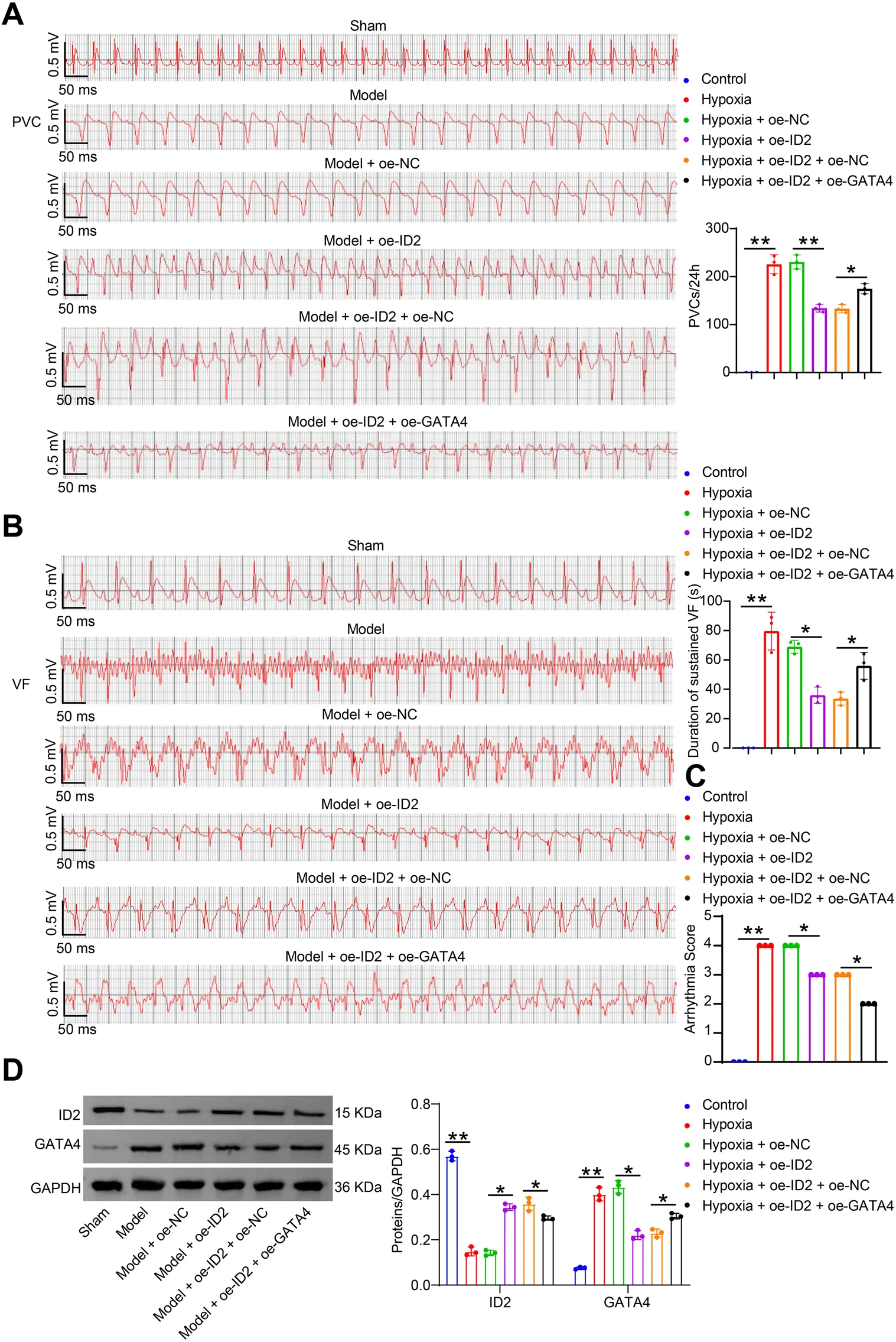

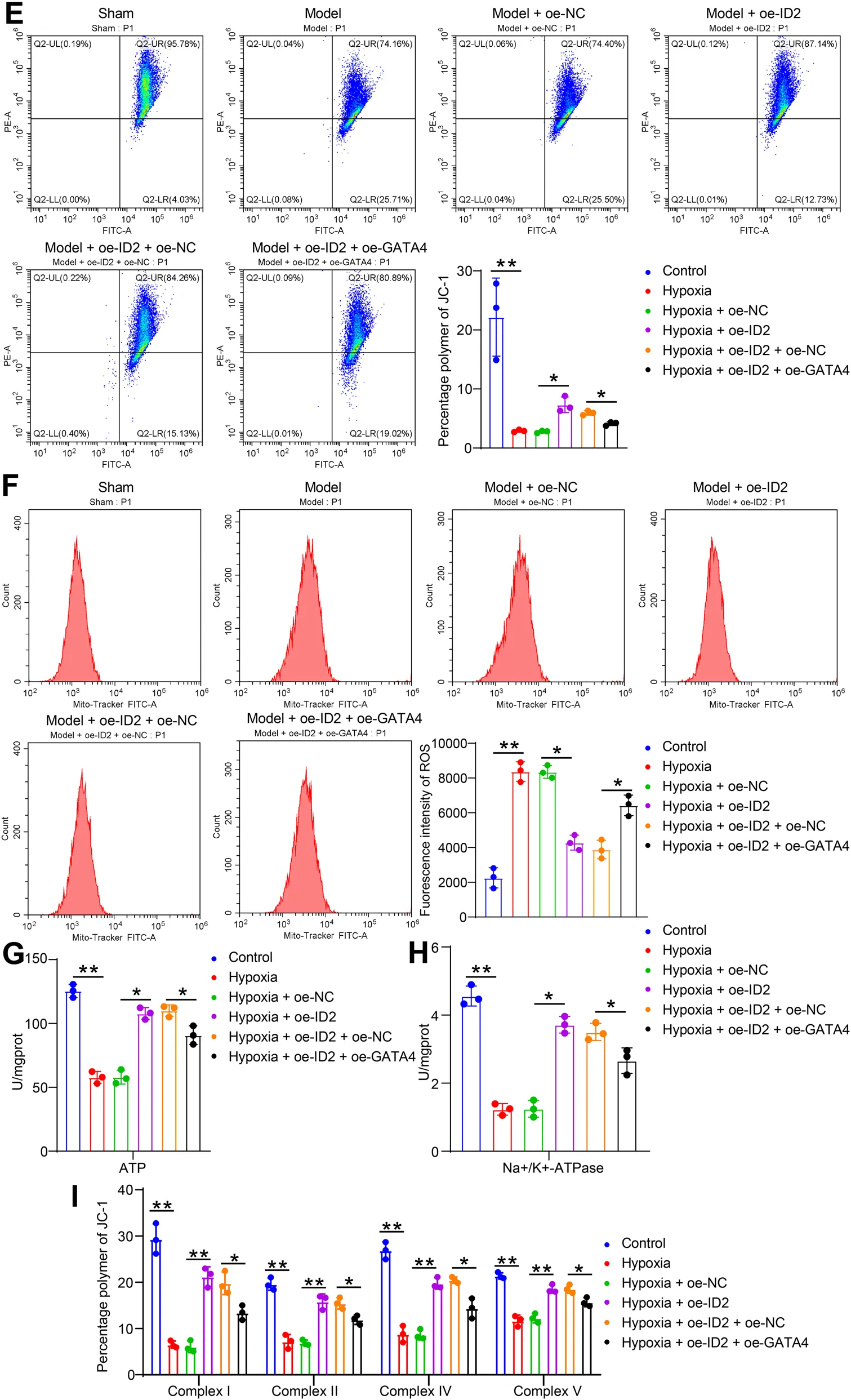
Overexpression of ID2 attenuates LAD-induced arrhythmias in MI rats via GATA4 downregulation. (A and B) ECG recordings and statistical analysis of PVCs and VF duration in rats. (C) Arrhythmia scoring in rats. (D) Western blot detection of ID2 and GATA4 expression in myocardial tissues. (E) Detection of mitochondrial membrane potential. (F) Detection of ROS in mitochondria. (G) Detection of ATP synthesis. (H) Na^+^/K^+^-ATPase activity assay. (I) Detection of Complex I, Complex II, Complex IV, and Complex V levels. Data are represented as the meanᵒ±ᵒSD, n=3 rats per group, representing biological replicates. Statistical significance was determined using one-way ANOVA with Tukey’s multiple comparisons test. *p<0.05, **p<0.01.

Next, the *in vitro* findings were further validated *in vivo*. Rats in the Model group demonstrated pronounced arrhythmogenesis, including frequent PVCs and VF. Strikingly, ID2 overexpression markedly attenuated arrhythmia severity, manifesting as reduced PVC frequency, shortened VF duration, and decreased arrhythmia scores compared with the Modelᵒ+ᵒoe-NC group. Conversely, concurrent GATA4 overexpression in the Modelᵒ+ᵒoe-ID2ᵒ+ᵒoe-GATA4 group reversed the ID2-mediated protective effects and exacerbated arrhythmia severity, as evidenced by increased PVC frequency, prolonged VF duration, and elevated arrhythmia scores, thereby confirming GATA4 as a downstream target of the antiarrhythmic action of ID2 (Figure 8A–C). Myocardial tissues from Model rats showed decreased ID2 and elevated GATA4 expression. ID2 overexpression suppressed GATA4 levels without affecting ID2 expression, whereas GATA4 overexpression restored GATA4 expression in the presence of oe-ID2 (Figure 8D). Additionally, GATA4 overexpression in MI rats reduced mitochondrial membrane potential and ATP levels, increased ROS production (Figure 8E–G), and suppressed the activities of Complexes I, II, IV, and V, as well as Na^+^/K^+^-ATPase activity. The inhibitory effects of oe-ID2 on mitochondrial dysfunction and Na^+^/K^+^-ATPase dysregulation were reversed by oe-GATA4 (Figure 8H and I). These results demonstrate that ID2 preserves mitochondrial function and ion homeostasis, thereby alleviating LAD-induced arrhythmias in MI rats by downregulating GATA4.

## Discussion

VA represents a major complication in patients with MI [31]. However, treatment options for VA remain limited, and elucidating the underlying molecular mechanisms may facilitate the development of novel therapeutic approaches. In this study, we established an MI rat model via LAD coronary artery ligation and identified significantly downregulated ID2 expression. Further *in vivo* and *in vitro* experiments were conducted to investigate the role of ID2 in post-MI VA and its potential molecular regulatory mechanisms. Our results demonstrated that ID2 attenuated mitochondrial dysfunction, Na^+^/K^+^-ATPase activity disturbance, and LAD-induced VA by suppressing GATA4 expression, thereby revealing the protective role of ID2 in post-MI VA and its regulatory relationship with GATA4.

Transcriptional regulator ID2 is an important gene involved in developmental processes, and its expression level within cells is critical for maintaining normal physiological functions [32]. In addition to its role in developmental regulation, ID2 also plays a significant role in tumorigenesis, such as promoting the proliferation of thyroid cancer cells [33]. In the cardiac context, ID2 exerts a key regulatory function in embryonic cardiac development [13]. In the present study, we found that ID2 expression was significantly downregulated in the myocardial tissue of LAD-induced MI rats, while overexpression of ID2 effectively attenuated post-MI arrhythmias, suggesting that ID2 may exert a protective role in the development and progression of VA and may hold potential as a therapeutic target. Notably, Erdogan et al. demonstrated that Gal-3 levels exceeding a specific threshold could effectively identify VA requiring clinical intervention, indicating its potential value as a predictive biomarker [34]. Similarly, as an important disease-related target, the expression level of ID2 in myocardial tissue may aid in assessing susceptibility to VA and therefore may possess certain predictive potential.

Mitochondrial dysfunction is a critical factor in cardiovascular pathogenesis [35]. It has been reported that targeting mitochondria enhances the therapeutic efficacy of nanomedicines for cardiac conditions [36]. In male MI mice, alleviation of vagal nerve dysfunction is closely associated with improvement of mitochondrial dysfunction [37]. Joseph et al. further reported that mitochondrial dysfunction serves as a critical predisposing factor for arrhythmogenesis [38]. When mitochondria are damaged, ROS production increases, while mitochondrial membrane potential and ATP levels decline significantly [35]. Mitochondrial dysfunction may also impair Na^+^/K^+^-ATPase activity, thereby compromising cellular function and even inducing cell death [39]. On the other hand, inhibition of Na^+^/K^+^-ATPase activity can enhance myocardial contractility [40]. Consequently, a deeper understanding of mitochondrial function regulation may provide novel insights for improving VA [41]. In this study, we observed that ID2 overexpression significantly alleviated mitochondrial dysfunction and restored Na^+^/K^+^-ATPase activity in hypoxic cardiomyocytes.

GATA4, a cardiac transcription factor, can reprogram cardiac fibroblasts into induced cardiomyocytes through its overexpression [42]. Studies have shown that GATA4 influences cardiomyocyte proliferation, and its mutations are associated with the development of cardiomyopathy [43], 44]. Moreover, GATA4 synergizes with TBX5 to promote cardiac development [45] and may also modulate sodium channel genes implicated in arrhythmogenesis [30]. To investigate the molecular mechanism of ID2 in VA, we predicted the interacting partners of ID2 and further confirmed that ID2 interacts with GATA4, which led us to select GATA4 as a downstream target of ID2 in VA pathogenesis. It has been reported that male rats are more susceptible to cardiac alternans and exhibit higher diastolic blood pressure compared with females [46]. In male HFpEF cardiomyocytes, Ca^2+^ handling abnormalities and electrophysiological changes are consistent with diastolic dysfunction and arrhythmic phenotypes, whereas in females, the mechanisms underlying aggravated diastolic dysfunction appear to depend more on alterations in myofilament properties [46]. In addition, related studies have demonstrated that male C57BL/6J mice show higher susceptibility to arrhythmias [47], further supporting the advantage of using male animals in arrhythmia model construction. Accordingly, we selected male rats for establishing the MI model. Our experimental results revealed that GATA4 was highly expressed in MI rats, while knockdown of GATA4 alleviated mitochondrial dysfunction and Na^+^/K^+^-ATPase dysregulation in hypoxic cardiomyocytes.

It has been reported that GATA4 and Nkx2.5 can induce the promoter activity of *Id2* and promote ID2 protein expression [48]. Furthermore, Ding et al. demonstrated that ID2 can directly bind to GATA4 and suppress the synergistic transactivation mediated by GATA4 and Nkx2.5 [49]. In addition, GATA4 has been shown to enhance ID2 protein stability by inhibiting its ubiquitination [49]. In contrast, our experimental results revealed that ID2 negatively regulates GATA4 expression and that overexpression of ID2 alleviates VA in MI rats by suppressing GATA4. Collectively, these findings suggest the existence of a complex bidirectional regulatory relationship between ID2 and GATA4. We speculate that under normal physiological conditions, such as in differentiated cardiomyocytes, GATA4 may act as a positive activator of ID2 expression. However, under pathological conditions, such as post-MI arrhythmia, aberrant GATA4 expression may trigger an ID2-mediated negative feedback regulatory mechanism to restrain excessive GATA4 activation.

Notably, the ID2-GATA4 regulatory axis is not an isolated molecular interaction but rather may serve as a functional upstream signaling node that governs mitochondrial homeostasis. Studies have shown that under pathological conditions such as MI, aberrant upregulation of GATA4 can mediate adverse ventricular remodeling and cardiomyocyte dysfunction [50]. Mitochondrial dysfunction disrupts the electrophysiological stability of cardiomyocytes and represents a critical driving factor for VA [51]. Multiple endogenous regulatory pathways have been reported to suppress post-ischemic arrhythmias; for instance, inhibition of ANXA2-mediated mitochondrial function alleviates cardiac injury after MI [52], and suppression of USP38-regulated inflammatory pathways attenuates early-stage inflammatory responses [53]. Consistent with these protective regulatory patterns, the present study demonstrates that ID2 ameliorates mitochondrial dysfunction and restores cardiomyocyte electrophysiological homeostasis by suppressing pathological GATA4 signaling, thereby attenuating ischemia-induced VA. These findings provide preliminary evidence for the functional link between ID2 and GATA4. Although the specific molecular mechanism by which ID2 regulates GATA4 remains to be further elucidated – which constitutes one of the limitations of this study. Overall, our work establishes a causal cascade linking the ID2-GATA4 axis, mitochondrial function, and VA, further refining the mechanistic framework underlying post-MI VA.

Studies have shown that Zhang et al. improved cardiac function in mice with arrhythmogenic cardiomyopathy through AAV9-mediated delivery of the PNPLA2 gene [54], suggesting that AAV-mediated delivery of ID2 or GATA4-related genes may be feasible for VA therapy. However, AAV9-mediated gene therapy still faces challenges such as immunogenicity and interspecies variability, which limit its feasibility for widespread clinical application in the near term [55]. Moreover, the clinical translation of AAV is constrained by high production costs and low transduction efficiency [56], and high-dose AAV administration may induce adverse effects, including hepatic and renal dysfunction, while the durability of its therapeutic efficacy also requires further validation [57], 58]. Therefore, at present, AAV9-mediated gene therapy still encounters numerous obstacles in clinical translation. Heo et al. demonstrated that apigenin, a non-toxic dietary flavonoid, enhances ID2 expression and thereby suppresses bladder cancer cell proliferation [59]. However, the oral bioavailability of apigenin is relatively low, which poses challenges for its clinical application [60]. Thus, although small-molecule drug strategies offer a new pathway for developing safe ID2-targeted antiarrhythmic therapies, issues such as bioavailability and targeted delivery efficiency still warrant further investigation and optimization in future studies.

This study had some limitations. Rotenone has been reported to induce gut microbiota dysbiosis and contribute to the pathological process of Parkinson’s disease, and it has also been shown to promote osteosarcoma progression through the induction of ROS release [61], 62], indicating its pleiotropic effects. Digoxin also possesses certain toxicity, and its serum concentration is positively correlated with the risk of mortality in patients with heart failure [63]. Given the complex *in vivo* toxicity and side-effect profiles of these two agents, we did not perform *in vivo* intervention experiments in this study, which to some extent diminishes the clinical translational value. In future studies, we will consider screening Complex I inhibitors and Na^+^/K^+^-ATPase inhibitors with better safety profiles that are suitable for *in vivo* application, and further evaluate their efficacy and mechanisms at the whole-animal level.

In conclusion, this study identifies dysregulated ID2 and GATA4 expression in post-MI VA and preliminarily elucidates a potential mechanism of ID2 in VA, namely that ID2 interacts with GATA4 to preserve mitochondrial function in cardiomyocytes, thereby maintaining Na^+^/K^+^-ATPase activity and attenuating post-MI VA. Our findings provide additional insight into the molecular mechanism of VA and identify a potential therapeutic target for its treatment.

## Supplementary Material

Supplementary Material
