# Supplementary material for: ID2 attenuates post-MI ventricular arrhythmias by targeting GATA4 suppression to preserve mitochondrial function and Na+/K+-ATPase activity
Source: Open Med (Wars). 2026 Sep 22;21(1):20261518. doi: 10.1515/med-2026-1518 (PMC13596124; doi:10.1515/med-2026-1518)
Supplement: Supplementary file 1 [file j_med-2026-1518_suppl_001.docx]

Supplementary Material

**Supplementary Figure S1.** **(A)** RT-qPCR and western blot analysis of ID2 transfection efficiency in cardiomyocytes. **(B)** Bright-field images showing freshly isolated neonatal rat cardiomyocytes and cells cultured for 24 hours. **(C)** Representative bright-field images of cells prepared for the detection of mitochondrial membrane potential and ROS by flow cytometry. (**D and E**) Representative flow cytometry gating plots of cardiomyocytes.
